# Young-Onset Cancers—Early Steps in the Right Direction

**DOI:** 10.3390/cancers15092599

**Published:** 2023-05-04

**Authors:** Savio George Barreto, Irit Ben-Aharon

**Affiliations:** 1 College of Medicine and Public Health, Flinders University, Adelaide, SA 5042, Australia; 2 Division of Surgery and Perioperative Medicine, Flinders Medical Center, Bedford Park, Adelaide, SA 5042, Australia; 3 Department of Surgery, Flinders Medical Centre, Bedford Park, Adelaide, SA 5042, Australia; 4 Division of Oncology, Rambam Health Care Center, Haifa 3109601, Israel; iritbenaharon@gmail.com; 5 Rappaport Faculty of Medicine, Technion, Haifa 3200003, Israel

The global incidence of young-onset (YO) cancer is on the rise. Determining the cause of this disturbing trend has been listed as one of the Grand Cancer Challenges [1]. The incidence of YO cancers in the gastrointestinal tract are on the rise in the past two to three decades, in the United States, with YO colorectal cancer increasing across the world [2]. To note, incidence rates of some countries may be partly due to the lack of a strict cancer registry of every documented case, and, therefore, while there is a lot of work to be done to address the problem, the first steps include advocacy and the need to encourage reporting of the burden of the problem. This may help identify trends that could guide focused research into the etiology. This Special Issue hosted by *Cancers* entitled “Young-onset GI (gastrointestinal) cancer” presented clinicians and researchers around the world with a platform to submit their research focused on YO cancer.

This Special Issue boasts seven peer-reviewed publications, including three from Europe and two each from the United States and Australia. ***La Pelusa and colleagues*** provided important insights into the burden of early-onset gastric cancer (EOGC) from the United States [3]. Their findings highlight an increased likelihood of EOGC affecting female patients and individuals who identified as Asian/Pacific Islander, African American, and Hispanic. They also note that patients with EOGC are more likely to be uninsured and to present with stage IV disease compared with their older counterparts. The variability in cancer care (surgery and chemotherapy) delivery presents opportunities for intervention if we wish to improve survival within this subset of patients. ***Schell and Shepherdson and their colleagues *** from Australia provided compelling data (over two manuscripts) on the trends in YO GI (oesophagus, stomach, pancreas, colon, and rectum) adenocarcinomas from South Australia and the Northern Territory (of Australia) over the last 28 years [4,5]. Although the rising trends in YO GI adenocarcinomas in South Australia, especially amongst males, appear to attract one’s attention, the existing high incidence for all YO GI adenocarcinomas in the Northern Territory, despite being unchanged over the study period, signals a worrying statistic that certainly warrants further investigation. The incidence rates noted for pancreatic cancer mirror the values seen in younger Black and Hispanic women in the United States [6]. ***Shepherdson* et al**. also addressed the incidence and survival rates amongst the Indigenous peoples (Aboriginal and Torres Strait Islander peoples) of Australia living in the Northern Territory [5]. The significantly lower survival compared with non-Indigenous peoples highlights an important area for health advocacy and the need for culturally safe Indigenous community-focused programs aimed at early detection and patient-centered management of GI adenocarcinomas. ***Ten Kate and colleagues*** from the Netherlands and Finland provide preliminary evidence on the susceptibility of the oesophageal epithelial homeostasis to acidic disturbances in individuals born with oesophageal atresia linking this observation to the increased propensity of this cohort of patients to develop early-onset Barrett’s oesophagus [7]. ***Pocurull and colleagues*** from Spain performed a germinal genetic analysis on patients with EOGC [8]. They note that familial aggregation was observed in only 15% of cases, whilst a germline mutation was found in 25% of patients tested with clinical criteria. Their findings are important in terms of highlighting the genetic heterogeneity of EOGC thereby reinforcing the need for accurate genetic counseling as well as enhancing the emerging use of multigene panels.

The issue also features two review articles by ***Castle and colleagues*** [9] on paediatric neuroendocrine neoplasms and ***Dudzisz-Śledź and colleagues*** on the treatment of gastrointestinal stromal tumours in younger patients [10].

The aetiology of YO cancers remains to be clarified. Ben-Aharon et al. [11] recently provided an up-to-date review of early-onset GI cancers. Due to the fact the majority of these cases are sporadic, the aetiological factors imply a key role in environmental factors. If this is so, then why are we seeing a changing trend only amongst the young over the last few decades? The PELICan hypothesis [12,13] may help reconcile these differential effects of the same triggers (for carcinogenesis) on different individuals. Another study recently published in Nature [14] provides insight into the germline mutation rates in vertebrates, including humans. The rising trend in the incidence of YO cancers in males may be linked to the finding of Bergeron et al. [14] that per-generation mutation rates are much higher in the males of a species. So, how then can we explain the higher than usual YO cancer rates noted in younger females only within some racialised groups [3,6]? Shirazi and Rosinger [15] determined that non-Hispanic (NH) African American and Hispanic girls have a significantly lower age of menarche by about 4.3 (SE = 0.08, *p* < 0.001), and 3.2 months (SE = 0.09, *p* < 0.001), respectively, relative to NH white girls. Bergeron et al. [14] found that age at maturity and species-level fecundity are the key life-history traits affecting germline mutation variation among species. These hypotheses may explain the differential effect that aetiological factors may have on individuals to increase their risk of developing YO cancers.

Clearly, there is work to be done to improve the early detection and multi-disciplinary management of YO cancers. We remain hopeful that this Special Issue in *Cancers* will serve its purpose of advocating for action because our young people need our help.
